# Deep cascaded multitask framework for detection of temporal orientation, sentiment and emotion from suicide notes

**DOI:** 10.1038/s41598-022-08438-z

**Published:** 2022-03-15

**Authors:** Soumitra Ghosh, Asif Ekbal, Pushpak Bhattacharyya

**Affiliations:** Department of Computer Science and Engineering, Indian Institute of Technology Patna, Patna, 801103 India

**Keywords:** Computational models, Machine learning, Computer science

## Abstract

With the upsurge in suicide rates worldwide, timely identification of the at-risk individuals using computational methods has been a severe challenge. Anyone presenting with suicidal thoughts, mainly recurring and containing a deep desire to die, requires urgent and ongoing psychiatric treatment. This work focuses on investigating the role of temporal orientation and sentiment classification (auxiliary tasks) in jointly analyzing the victims’ emotional state (primary task). Our model leverages the effectiveness of multitask learning by sharing features among the tasks through a novel multi-layer cascaded shared-private attentive network. We conducted our experiments on a diversified version of the prevailing standard emotion annotated corpus of suicide notes in English, CEASE-v2.0. Experiments show that our proposed multitask framework outperforms the existing state-of-the-art system by 3.78% in the Emotion task, with a cross-validation Mean Recall (MR) of 60.90%. From our empirical and qualitative analysis of results, we observe that learning the tasks of temporality and sentiment together has a clear correlation with emotion recognition.

## Introduction

Suicide has been a significant public health concern for decades, and one of the leading causes of death worldwide^1^. With around 800 thousand people committing suicide each year, it is now a global priority to control the ever-increasing rise in the rate of suicide. Suicide notes can provide valuable insights into the minds of deceased persons. Health experts investigate suicide notes with the hope that they can use this first-hand information to improve their understanding of the thinking patterns of the deceased persons that led them to commit suicide.

A suicide note content usually exhibits various sentimental and emotional facts and implications revolving the author of that note. Though closely related, there are firm distinctions between the connotations of *Sentiment* and *Emotion*^2^. *Emotion* associates to a mental feeling (example: disgust, fear or anger) while *Sentiment* expresses an opinion produced by that feeling about something. While *Emotion* can be classed as coarse-grained (Ekman’s fundamental emotions^3^ or fine-grained (Plutchik’s Wheel of Emotions^4^, *Sentiment* is commonly realised as a *positive* and *negative* score, referred to as polarities. A third assessment, *neutral*, denotes an occurrence that is neither *positive* nor *negative*.

In psychological research, Time perspective orientation (TPO) has been hypothesized to play an essential role in predicting negative psychological functioning and outcomes such as suicide risk. The way a person connects to the temporal concepts of past, present, and future, both behaviorally and psychologically, is referred to as the TPO^5^.

The current study considers an amalgamation of the concepts of *Time Perspective, Sentiment and Emotion* concerning the content of suicide notes. It investigates the role of the former two in aiding the process of emotion recognition. The following instances are sentences from real-life suicide notes:

- *I’ll make a good specimen cadaver to the future doctors of India.*
- *My first memories as a child are of being raped, repeatedly.*

The first sentence expresses a feeling of *hopefulness* and is future-oriented, whereas the second sentence shows traces of *abuse* and connects to past temporal orientation. Similarly, certain emotions or feelings like *hopefulness, instructions, thankfulness* connect to present or future time perspective, whereas emotions like *abuse, guilt, blame* primarily relate to past temporal orientation. This association of various emotions of different polarities with varying time perspectives may help learn the underlying emotional feeling conveyed in the content of suicide notes.

In this work, we build an end-to-end system for detecting temporal orientation, sentiment, and emotion classes from sentences in suicide notes. Our model uses a shared-private attention framework that allows retaining both the global features (common to all the tasks) and task-specific features while making predictions at the output layer. Furthermore, we perform a multi-layer cascading of features that allows intermediate features from a previous sub-network to flow in the current sub-network. We consider the extended version of the benchmark emotion annotated dataset of suicide notes, *CEASE-v2.0*^6^, which contains 2539 additional sentences than the initially introduced version of the same dataset^7^. The dataset contains sentence-wise annotations for multi-label emotion classes (Emotion labels: *forgiveness, happiness_peacefulness, love, pride, hopefulness, thankfulness, blame, anger, fear, abuse, sorrow, hopelessness, guilt, information, instructions*). We employ *weak supervision* to generate sentiment labels *(positive, negative, neutral)* and temporal categories *(past, present, future)*.

We compare our proposed multitask system to task-specific single-task models and multiple multitask baselines created using state-of-the-art multitask architectures. On the emotion recognition task, our best model achieves a cross-validation Mean Recall (MR) of 60.90%, which is 4.43% higher than the most recent state-of-the-art model on a similar task.

The main contributions of our proposed work are summarized below:

- This is a first cross-sectional study involving simultaneously estimating time perspective, sentiment, and emotion on suicide notes.
- We employ weak supervision-based approaches for automatically annotating each instance in the dataset with temporal orientation and sentiment label.
- We present a multitask learning framework based on a shared-private attention network and model cascade that achieves improved overall performance for the emotion detection and sentiment classification task on suicide notes.

The remainder of the paper is organised as follows. The “Related work” section summarises some of the prior efforts in this area. Following that, in the “About dataset” section, we go over the dataset description and annotations in detail. We discuss our suggested technique for multitask experiments in “Proposed methodology” section. The next “Experiments, results, and analysis” section discusses the experiments done and their results. Finally, in the “Conclusion” section, we end our work and identify the scope of future study.

## Related work

The study of suicide notes dates back to the late 1950s when many researchers^8, 9^ studied the content of suicide notes to evaluate the many inferred motives for suicides. Ho et al.^9^ stated that the significance of a suicide note is explicitly realized when we investigate the context concerning the occurrence of suicide. Out of the 224 notes considered in this study, it was found that most of the note-leavers were young women and young people tend to write more extended notes than older adults. Several studies^10, 11^ were conducted to distinguish between fake and real suicide notes. The socio-economic and psychological variables of suicides were studied by Shneidman et al.^12^.

Laghi et al.^13^ found that time perspective has a relevant role to play in predicting suicidal ideation among adolescents. In a recent study, Åström *et al.*^14^ demonstrated a significant relationship between changes in time perspective with shifts in age interval. People tend to stay worried about their future (future negative) during young age while their state of worry is focused on their present (present fatalistic) situation with increased age. On similar lines, Lefèvre et al.^15^ studied the differences in time perspective among depressed and non-depressed patients and observed various alterations in the time perspective of depressed patients when compared to non-depressed persons.

Several machine learning-based models were proposed^16, 17^ to detect real suicide notes from the simulated ones, which were able to outperform the classification accuracies attained by human annotators. It is demonstrated in literature^18^ that machine learning and Natural Language Processing (NLP) approaches serve great potential for detecting externally verified suicide risk from daily social media activity. The inclusion of theory-driven elements when employing such systems is critically significant. Fernandes et al.^19^ provided an NLP-based rule-based technique to classifying suicidal thoughts, as well as a hybrid machine learning and rule-based strategy to detecting suicidal attempts in a psychiatric clinical database. To assist clinical researchers in understanding and managing communications in social media, Gkotsis et al.^20^ investigated the ability of a social media platform in automatically classifying mental health-related information. The authors designed automated deep learning architectures to detect mental health-related Reddit posts (binary classification) and then categorize them according to subreddit groups based on themes (multiclass classification). Emotion plays an important role in developing competent and robust Artificial Intelligence (AI)-based systems^21, 22^. Akhtar et al.^22^ presented an end-to-end deep neural network for detecting emotion, sentiment, and intensity values across a variety of issues and domains. In a following work by the authors^21^, a deep-stacked model was developed to predict the intensity of emotion or sentiment felt by a Twitter user, given the tweet and the associate emotion or sentiment label. Researchers have not fully studied the impact of cognitive function in suicidal thoughts in individuals with major depressive disorder (MDD). A correlation between cognitive performance and suicidal thoughts was examined by Pu et al.^23^ which was conducted on MDD patients. Suicidal ideation and performance of other mental orders detection were improved using relation network-based attention mechanism in work by Ji *et al.*^24^.

Pestian et al.^25^ presented in track 2 of the 2011 i2b2 NLP Challenge a huge emotion annotated corpus of 900 suicide notes in English to assist study and innovation in emotion identification and suicide prevention. This dataset facilitated the development in many facets^26–29^ from a classification perspective using the various NLP techniques^30^. Each instance in the dataset is annotated with a single emotion from a fine-grained 15 emotion classes, which is also adapted by Ghosh et al.^7^ to label the CEASE-v2.0 corpus. However, the present unavailability of the i2b2 corpus of suicide notes led to the development of the CEASE-v2.0 dataset to bridge the gap. The authors in their following work^6^ extended the CEASE-v2.0 corpus with additional 2539 instances annotated with multi-label emotion labels and also annotated the extended dataset with depression labels and sentiment labels.

Many recent studies have introduced novel methodologies to address various topics on sentiment analysis from diverse application areas. Yadav et al.^31^ studied people’s feelings in the top five nations most impacted by the new coronavirus, and created a COVID-19 Sentiment Dataset of tweets. The scientists also suggested a Multilevel Attention-based Conv-BiGRU network (MACBiG-Net) for sentiment classification that catches subtle cues in a document by concentrating on local text properties as well as past and future context information. Using the Stacked LSTM model and Stanford’s pretrained GloVe embeddings, Saini et al.^32^ investigated the psychometric influence of the COVID-19 infodemic. The purpose of this research was to present a unique application for identifying hidden sentiment in microblogs. Yadav et al.^33^ presented a deep affect-based movie genre identification system based on extracting emotional material from movie trailers. During the process, the scientists produced EmoGDB, an affect-based dataset containing 100 Bollywood movie trailers annotated with different Bollywood genre-specific emotions classes. Yadav et al.^34^ investigated the usefulness of sentiment analysis in the medical industry by reviewing patient evaluations of well-known medications. Based on TF-IDF and FastText word embeddings, the authors suggested a weighted text representation approach. The suggested strategy beat several metrics’ selected baselines on the medication review dataset, according to the results.

Yadav et al.^35^ provides a comprehensive summary of the most major bio-inspired algorithms utilised in sentiment analysis. The authors reviewed eighty publications from journals, conferences, book chapters, and other sources to address the state-of-the-art on these significant algorithms and to conduct a comparative study on these algorithms. Agarwal et al.^36^ proposed four RNN variants for assessing the utterances of the speakers from the videos: GRNN, LRNN, GLRNN, and UGRNN, which achieves superior sentiment classification accuracy on individual modality (text, audio, and video) than existing algorithms on the CMI-MOSI dataset, as demonstrated by experimental results on the same dataset. Yadav et al.^37^ studied visual sentiment analysis using a residual attention-based deep learning network (RA-DLNet). The authors leveraged a residual attention model that focused on the image’s sentiment-rich local regions as the image’s local areas communicate strong feelings. The efficacy of the proposed approach was demonstrated by a comparison of accuracy with similar state-of-the-art. XRANet (Xception Residual Attention-based network), a deep learning-based architecture for visual sentiment analysis, was proposed by Yadav et al.^38^. The XRA-Net architecture’s effectiveness was assessed using the publicly accessible, real-world Twitter-I dataset. Obtained results indicated that the suggested architecture outperformed all previous findings.

Recent works^39–41^ have shown the effectiveness of multitasking systems by learning several correlated tasks simultaneously. Majumder et al.^39^ developed a deep neural-based system to detect sentiment and sarcasm in a multitask setting and observed that performance of both the tasks improve when learned upon jointly. In work by Qureshi et al.^40^, an attention-based neural network was trained on multimodal inputs (audio, video, and text) to address the tasks of depression detection and its intensity prediction. The work by Akhtar et al.^41^ on the development of a multitask system to detect emotion, sentiment, and intensity values for several problems and domains have motivated us to build one of our baselines in this study.

Because of the sensitivity and stigma connected with any suicide deed, the public availability of suicide notes is relatively limited. Track 2 of the 2011 i2b2/VA/Cincinnati Medical Natural Language Processing Shared task^25^ introduced a huge emotion annotated corpus of phrases derived from 900 English suicide notes. However, the corpus was later discontinued for usage due to privacy concerns. Other related corpora are either very small in size^42, 43^ or are not essentially developed from genuine suicide notes^44, 45^. The Northern Ireland Suicide Study^42^ comprises a compilation of data from a range of documentation sources, including coroners’ files, including suicide notes. Out of the 118 suicide studied in this work, suicide notes were available for 42 notes only^46^. On similar lines, McClelland *et al.*^43^ hypothesized that rather than looking for the underlying psychiatric reasons for suicide in the content of suicide notes, they could be looked at as a mode of communication that help to control the blame assigned to both the author and the receivers of the suicide note. This study analyzed 172 suicide notes left by 120 suicide victims. At present, to the best of our knowledge, the CEASE-v2.0 corpus introduced by Ghosh et al.^6^ is the largest available suicide notes corpus publicly available for research purposes. The dataset is created from over 350 real-life suicide notes with 4932 sentences and manual annotations for multi-label emotions and depression labels. Furthermore, the emotion annotations are available for 15 fine-grained emotions which have been considered in prior studies^25, 44^ on suicidal research. This motivated us to consider the extended version of the CEASE-v2.0 dataset that was introduced by Ghosh et al.^6^ as it contained approximately double the instances than the original version introduced in the introductory work^7^ by the same authors. In addition, the authors investigated the impact of depression and sentiment in boosting emotion detection ability from suicide notes in a multitask scenario. This allows us to assess our model’s efficacy by comparing its performance to that of this state-of-the-art system. We develop numerous more deep multitask classifiers for temporal categorization, sentiment detection, and emotion identification tasks and compare their performance to single-task equivalents.

## About dataset

In this work, we use the latest version of emotion annotated CEASE-v2.0 dataset (Resource available at: www.iitp.ac.in/~ai-nlp-ml/resources.html) released by Ghosh et al.^6^. This version of the dataset contains a total of 4932 instances, 2539 instances more than the initially introduced version^7^ of the same resource.

### Data collection

Similar steps and approaches as in^7^ were followed in^6^ for data collection and cleaning to minimize any nature and type variations that may arise due to the collection of data in two different phases. Credible sources like popular news bulletins, e-newspapers, blogs, etc., were considered while fetching any suicide note content. Images of handwritten notes were manually transcripted and digitized to their plain-text form, while pictures of typed notes were extracted using Optical Character Recognition (OCR).

### Data annotation

Three annotators performed the multi-label emotion annotations on each sentence of the extended CEASE-v2.0 dataset. Sentences were shuffled over the entire dataset, and various anonymization tags (NAME, ADDRESS, ORGANIZATION, TIME, DATE, SECTION_NOT_CLEAR, DELETED, SIGNATURE) were used to cover up information that may compromise the anonymity of a note or an individual. We generate the sentences’ temporal labels and sentiment classes using weak supervision. Some annotated sample instances are shown in Table 1.

**Table 1:** Sample annotations from the extended CEASE-v2.0 dataset.

Sentence	Temporal	Sentiment	1st Emo	2nd Emo	3rd Emo
I did have dreams, just like everyone	Past	Negative	Sorrow	Hopelessness	
Like cherry blossoms in the spring let us so pure fall and radiant	Future	Positive	Hopefulness	Happiness	Instructions
Wherever I look... they have come for me and I must go	Future	Neutral	Information		
And the way the current system is set up, it protects all cops whether good or bad, right or wrong, instead of punishing bad cops & holding them accountable for their actions	Present	Negative	Blame	Anger	Information

#### Multi-label emotion annotation

The authors in^6^ labeled each instance of the extended CEASE-v2.0 dataset with multi-label emotions (up to 3 emotions per instance) as opposed to the single-labeling scheme employed by^7^. This makes it possible to associate multiple emotion classes for a sentence and capture all the essential emotional traits. The emotion labels for a given sentence were marked in their order of predominance, i.e., the most expressive emotion in a sentence is marked as the *1st Emo*, followed by the next most expressive emotion, *2nd Emo* and similarly, the *3rd Emo* is also marked. The inter-rater agreement among the annotators was measured using Krippendorff’s $\alpha$ coefficient^47^ statistical measure and attained a score of 0.61, signifying that annotations were of good quality. Table 2 shows the count of instances annotated with one, two, and three emotion labels. We observe that 76% of sentences are annotated with a single emotion label, whereas 22% are annotated with two emotion labels. Only 2% of instances in the dataset are annotated with three emotion labels.

**Table 2:** Count of instances with single and multiple emotion labels.

Type	Count
One emotion label	3742
Two emotion labels	1070
Three emotion labels	120

#### Generation of temporal category labels using weak classifier

We train a neural network for the classification of temporal orientation on the gold-standard temporal orientation tweet corpus^48^ which contains 27k tweets (9k tweets in each temporal category). The neural network is built on top of pre-trained GloVe embeddings, followed by a Bidirectional Gated Recurrent Unit (BiGRU) encoding layer and a word attention layer with softmax activation in the output layer. The model achieved an accuracy of 75.36% on the test set, close to the reported accuracy of 78.27% in the base paper. We use this classifier to generate weak temporal labels for the instances in our extended CEASE-v2.0 dataset. To evaluate the quality of the predicted labels, we manually annotate 300 sentences randomly from our dataset and match our annotations with the predicted labels for the same 300 sentences. We observed a 0.84 Kappa score between our manual annotations and the weak labels, which depict that the predicted labels are of considerably good quality. Table 1 shows some sample instances from our dataset with predicted temporal categories.

Data preprocessing of temporal data Because the Time Perspective^48^ benchmark dataset is made up of tweets, we employ the text processing tool *ekphrasis*^49^, which was designed to handle postings from social media sites such as Twitter, Facebook, and others. Using word statistics from two large corpora, it can do tokenization, word normalisation, word segmentation, and spell correction (English Wikipedia, Twitter).

- *Normalization* We replace URLs, emails, phone, dates, etc. with the following normalized words: ’url’, ’email’, ’phone’, ’date’, etc.
- *Segmentation* exphrasis’ ’twitter’ corpus is used for word segmentation on hashtags (#BESTFRIENDS $\rightarrow$ best friends).
- *Contractions* We use exphrasis’ ’twitter’ corpus to unpack contractions (can’t $\rightarrow$ can not; i’m $\rightarrow$ i am).
- *Spell correction* Spelling is fixed for elongated words (Soooo $\rightarrow$ so; YOOOU $\rightarrow$ you).

#### Mapping emotion classes to sentiment polarities

Following the work in^6^, we categorize the instances of the dataset into three categories, signifying the three sentiment classes, *viz.* *Positive, Negative* and *Neutral*. The distinctive (minimal or no overlap) emotion classes in the 15 emotion tagset introduced by^25^ can be easily associated with at most one of the sentiment polarities. We map the emotion classes to their corresponding sentiment evaluations as follows:

- Emotion bearing Positive sentiment: *Forgiveness, Happiness_Peacefulness, Hopefulness, Love, Pride, Thankfulness*
- Emotions bearing Negative Sentiment: *Abuse, Anger, Blame, Fear, Guilt, Hopelessness, Sorrow*
- Emotions bearing Neutral sentiment: *Information and Instructions*

The primary emotion *(1st Emo)* being the predominant emotion for a sentence is considered for mapping to an equivalent sentiment category (refer to Table 1). This labeling method bears the minimal cost for generating noisy labels saving manual effort and time annotating the instances from scratch. We manually annotated 300 sentences randomly from our dataset with sentiment labels and observed a 0.74 Kappa score between our manual annotations and the weak labels, which depicts that the predicted labels are of considerably good quality.

Table 3 shows the data distribution over various temporal categories and sentiment classes.

**Table 3:** Data distribution over various temporal categories and sentiment classes.

Task	Classes		
Temporal	Past	Present	Future
	2300	792	1840
Sentiment	Positive	Negative	Neutral
	885	1500	2547

## Proposed methodology

**Figure 1:**
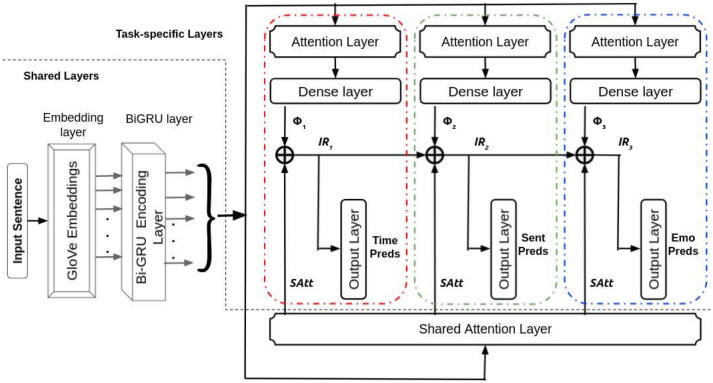
Architecture of shared-private attentive network with multi-layer cascades. Color-coded red, green, and blue dashed lines depict the three sub-networks participating in the layer cascading. The grey dashed line indicates the separation of the shared and task-specific layers.

To identify temporal orientation, sentiment, and emotion in suicide statements, we construct an end-to-end deep multitask system. All of the tasks are learnt together using an effective feature sharing network that makes use of shared and private attention features, as well as multi-layer cascades (subnets)^50^. The architecture of the suggested method is shown in Fig. 1.

### Problem definition

The goal of our multitask learning framework is to develop an automatic system for classifying a sentence S in: (a). one of the temporal categories *t*, where *t* $\epsilon$ {*past,* *present, future*}; (b). one of the sentiment categories *s*, where *s* $\epsilon$ {*positive, negative, neutral*}, and, (c). at most 3 emotion categories *e*, where *e* $\epsilon$ {*forgiveness, happiness*_*peacefulness, love, pride, hopefulness, thankfulness, blame, anger, fear, abuse, sorrow, hopelessness, guilt, information, instructions*}.

### Word embedding representation

To capture the syntactic and semantic information of the words in a phrase, we use the efficacy of a 300-dimensional pre-trained GloVe word embedding^51^ that is trained on the *Common Crawl* (42 billion tokens) corpus.

### Bidirectional GRU (BiGRU) encoding layer

The embedding layer’s word vectors are passed through a BiGRU^52^ layer (with 256 neurons) that captures contextual information from both the forward $(\overrightarrow{GRU})$ and backward $(\overleftarrow{GRU})$ timesteps, and produces hidden representations $(h_{\mathrm{it}})$ of each word in the sentence.

### Attention layer

We employ a word attention mechanism^53^ after the BiGRU layer to focus on the essential words in a sentence. The output from the BiGRU layer is fed to four identical attention layers, among which one is considered a shared attention layer, and the rest three are part of three private sub-networks (subnets). We discuss their details in the following section.

### Shared-private attentive network with multi-layer cascades (SPANMLC)

We apply the idea of model cascading by building an extensive network from a composition of 3 smaller networks (sub-networks, or simply, subnets). Rather than passing a particular output decision to the next subnet, we pass the output uncertainty (intermediate representation) to the next subnet. In a cascade method, the hidden layers of this network are connected as the inputs to the syntactic network (rather than the parts of speech prediction itself). Each subnet is associated with predicting the output for one particular task. The first subnet (color-coded red dotted lines) outputs Temporal Orientation predictions, the second subnet (color-coded green dotted lines) outputs Sentiment predictions, and the third subnet (color-coded blue dotted lines) results in emotion predictions. We order the subnets in the above order with the intuition that the sentiment task will be assisted with valuable temporal information. Since the tasks of sentiment and emotion are highly correlated, the subset corresponding to the emotion task will use this temporal-aided sentiment information to produce emotion-aware features.

The following equations can be used to represent the total flow of information via the network:

1 $$\begin{aligned} \Phi _{1}= & {} MLP_{1}(Att_{1}(BiGRU(x_1{\text {:n}}))) \end{aligned}$$

2 $$\begin{aligned} \Phi _{2}&= MLP_{2}(Att_{2}(BiGRU(x_1{\text {:n}}))) \end{aligned}$$

3 $$\begin{aligned} \Phi _{3}&= MLP_{3}(Att_{3}(BiGRU(x_1{\text {:n}}))) \end{aligned}$$

4 $$\begin{aligned} IR_{1}&= [\Phi _{1};SAtt] \end{aligned}$$

5 $$\begin{aligned} IR_{2}&= [\Phi _{2};SAtt;IR_{1}] \end{aligned}$$

6 $$\begin{aligned} IR_{3}&= [\Phi _{3};SAtt;IR_{2}] \end{aligned}$$

The output from the shared BiGRU layer $(BiGRU(x_1{\mathrm{:n}}))$ is fed to the three sub-networks, which have a private attention layer of their own followed by a dense layer which is a multi-layer perceptron (MLP_i_) or also known as fully-connected layer. The above BiGRU output is also fed to a shared attention layer whose output is (*SAtt*). Finally, we compute the intermediate representation (*IR*_i_) of each sub-network by linearly concatenating the outputs from that particular subnet’s dense layer ($\Phi$_i_), shared attention layer (*SAtt*) and the $IR_{\mathrm{i}}$ of the previous subnet. Each $IR_{\mathrm{i}}$ may be regarded of as an encoding that collects the necessary information for predicting a task’s outcome.

The overall architecture of the proposed approach is shown in Fig. 1.

*Calculation of loss* The following unified loss function is used to calculate the overall loss (*L*(*Omega*)) of the whole network:

7 $$\begin{aligned} \begin{aligned} L(\Omega ) = p * L1(IR_{1}(\Omega )) + q * L2(IR_{2}(\Omega ) | IR_{1}(\Omega )) + r * L3(IR_{3}(\Omega ) | IR_{2}(\Omega ), IR_{1}(\Omega )) \end{aligned} \end{aligned}$$,

L1, L2, and L3 are the three-loss terms corresponding to the three sub-networks. The gradient of a loss term includes the gradient of the current and preceding stages, according to the chain rule of backpropagation. All of the network parameters to be optimised are represented by *Omega*. The loss weights are p, q, and r.

#### Model parameters

We employ *dropout* of 0.25^54^ after each attention layer and dense layer in the network. The network is trained using Adam^55^ optimizer. The specifics of numerous hyper-parameters linked to our experiments are shown in Table 4. All of the experiments were carried out using a GeForce GTX 1080 Ti GPU.

**Table 4:** Details on numerous hyper-parameters used in our implementations.

Parameters	Details
Hidden activation	*ReLU* (dense layers)
Output activation	*Softmax* (temporal and sentiment tasks); *Sigmoid* (emotion task)
Batch size	32
Epochs	20
Dropout	0.25
Loss	*Categorical CrossEntropy* (temporal and sentiment tasks)
	*Binary CrossEntropy* (emotion task)
Loss weights	[0.3, 0.3, 1]
Optimizer	*Adam*

## Experiments, results and analysis

This section discusses the experimental setup and reports the experimental results and the necessary analysis.

### Experimental setup

At various phases of our implementations, we employ python-based libraries Keras and Scikit-learn^56^. We provide the results of the temporal orientation detection and sentiment categorization tasks in terms of accuracy and macro-F1 scores. We assess the emotion task’s performance using Mean Recall (MR) and samples-F1 (s-F1) measures.

We run stratified-kFold (k=10) cross-validation on our whole dataset for all experiments and present the mean scores for all metrics. Each cross-validation fold is made by retaining the percentage of samples for each emotion class constant, as the data is highly skewed over the various fine-grained emotion classes^6^. Since the value of k=10, 90% of the dataset is used for training for each fold of cross-validation, and the remaining 10% is used for testing.To handle the impact of noisy generated labels from the weak-supervision approaches, we weigh the losses from the 1st two subnets lesser than that from the emotion subnet. We apply Grid Search method to obtain the combination of loss weights, [0.3,0.3,1], and set them using the *loss_weights* parameter of Keras *compile* function.

### Baselines

#### Single-task systems

We build single-task deep learning systems for each of the three tasks wherein for each model, the BiGRU output is passed through the following sequence of layers: Attention layer, Dense layer, and a task-specific output layer.

#### Cascaded multitask system with external knowledge infusion (CMSEKI)^6^

Using SenticNet’s IsaCore and AffectiveSpace vector-spaces, the creators of^6^ created a deep multitask architecture with a knowledge module that adds common-sense information into the learning process. Using SenticNet’s IsaCore and AffectiveSpace vector-spaces, the authors of^6^ developed a deep multitask architecture with a knowledge module that adds common-sense information into the learning process. For our study, we used the dataset presented in this paper. At the same time, the system models emotion recognition (the primary goal), depression detection, and sentiment categorization (secondary tasks). The secondary tasks increased the effectiveness of the primary task when both tasks were learned concurrently.

#### Multitask 1^41^

Following the work of^41^, we develop a basic multitask system with a shared BiGRU layer (256 units) and an Attention layer and task-specific dense layers and output layers. The following equations represent the total flow of information via the network:

8 $$\begin{aligned} \Phi _{{1}}= & {} MLP_{{1}}(SAtt(BiGRU(x_1{\mathrm{:n}}))) \end{aligned}$$

9 $$\begin{aligned} \Phi _{{2}}= & {} MLP_{{2}}(SAtt(BiGRU(x_1{\mathrm{:n}}))) \end{aligned}$$

10 $$\begin{aligned} \Phi _{{3}}= & {} MLP_{{3}}(SAtt(BiGRU(x_1{\mathrm{:n}}))) \end{aligned}$$

**Figure 2:**
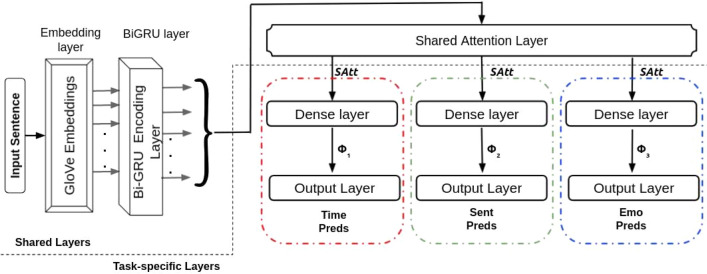
Architecture of basic multitask system with distinct shared and task-specific layers. Colour coded red, green and blue dotted lines depicts the three independent task-specific sub-networks.

A shared attention layer receives the output from the shared BiGRU layer $(BiGRU(x_1{\mathrm{:n}}))$. Before being routed to different output layers, the attention layer’s output (*SAtt*) passes through three task-specific dense layers. The general design of the proposed framework is depicted in Fig. 2.

*Calculation of loss* The framework’s cumulative loss $(L(\Omega ))$ can be calculated using the following unified loss function:

11 $$\begin{aligned} L(\Omega ) = p * L1(\Omega ) + q * L2(\Omega ) + r * L3(\Omega ), \end{aligned}$$

L1, L2, and L3 are the three-loss terms corresponding to the three sub-networks. $\Omega$ is the optimization parameters of the network.

#### Multitask 2^57^

Unlike passing the IR of a subnet to the following subnet (as in Fig. 1 (of the main file)), we instead allow the output of a subnet to flow to the next as in the work by^57^. The architecture is Multitask 2 is similar to our proposed approach (SPANMLC) except that the intermediate representation (IR_i_) from a subnet is not dependent on the IR of the previous subnet but the actual output of the previous subnet. We first obtain $\Phi _{{1}}$, $\Phi _{{2}}$ and $\Phi _{{3}}$ following the equations (1), (2) and (3) respectively. The intermediate representations, IR_1_, IR_2_ and IR_3_, are realized by the following equations:

12 $$\begin{aligned} IR_{{1}}= & {} [\Phi _{{1}};SAtt] \end{aligned}$$

13 $$\begin{aligned} IR_{{2}}= & {} [\Phi _{{2}};SAtt;O_{\mathrm{T}}] \end{aligned}$$

14 $$\begin{aligned} IR_{{3}}= & {} [\Phi _{{3}};SAtt;O_{\mathrm{S}}] \end{aligned}$$

**Figure 3:**
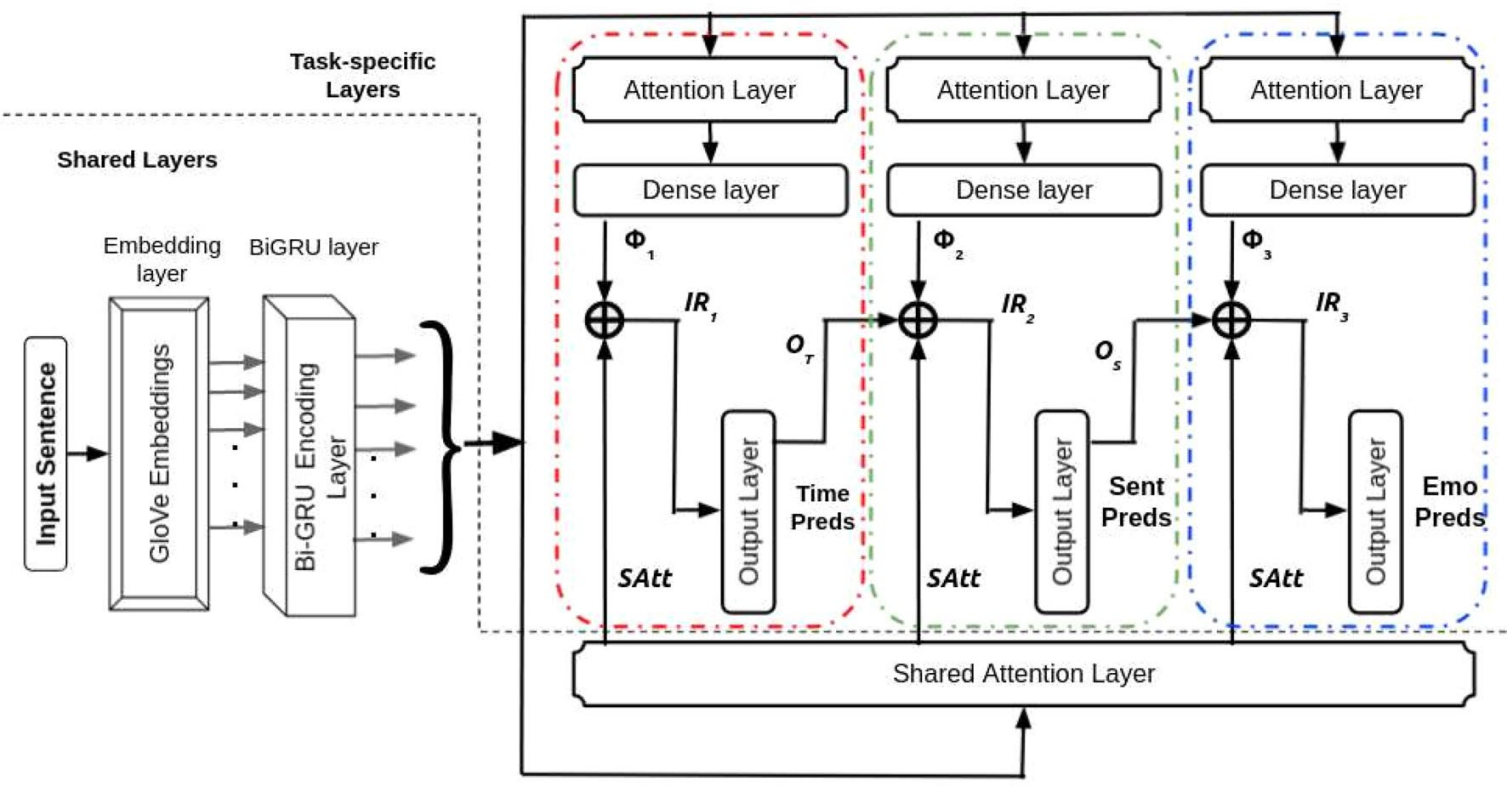
Architecture of shared-private attentive network with multi-layer cascades. Colour coded red, green and blue dotted lines depicts the three sub-networks participating in the layer cascading.

We compute the intermediate representation (IR_i_) of each sub-network by linearly concatenating the outputs from that particular subnet’s dense layer ($\Phi _{\mathrm{i}}$), shared attention layer (*SAtt*), and the output from the previous subnet (*O*). Figure 3 depicts the overall architecture of the suggested framework.

*Calculation of loss* The framework’s total loss $(L(\Omega ))$ can be realized using the same loss function as SPANMLC.

#### Multitask 3

To investigate the impact of multi-network cascades, we remove the private attention layers from the subnets in Fig. 1, keeping the rest of the architecture intact. We remove the private attention layers from the subnets in SPANMLC to build the architecture for Multitask 3. The idea is to learn the impact of multi-network cascades in independence in the proposed approach. The task-specific dense layer outputs, $\Phi _{{1}}$, $\Phi _{{2}}$ and $\Phi _{{3}}$, are produced following the equations (8), (9) and (10) respectively. The intermediate representations, IR_1_, IR_2_ and IR_3_, are realized following the equations (4), (5) and (6) respectively.

**Figure 4:**
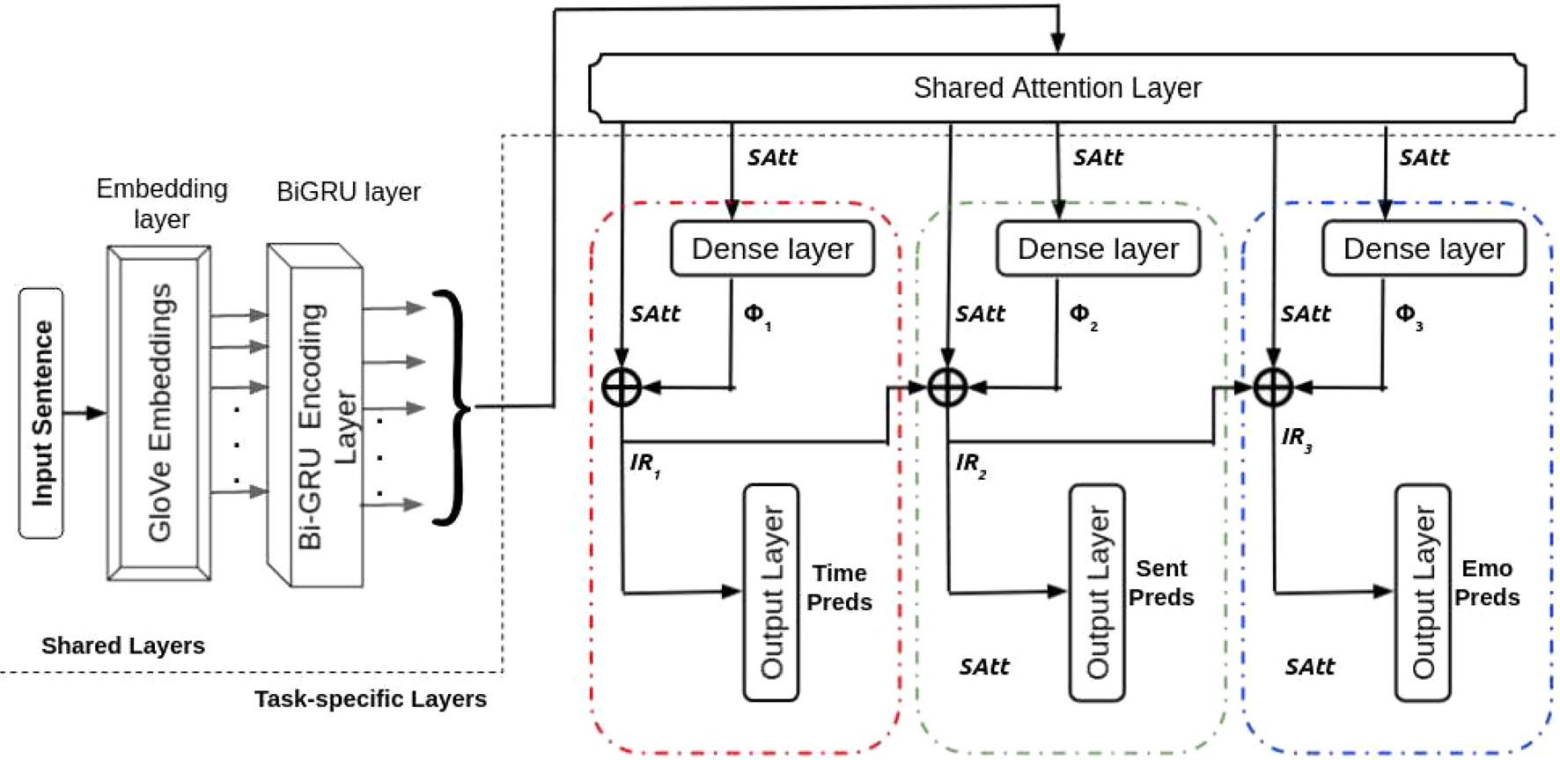
Architecture of shared attentive network with multi-layer cascades. Colour coded red, green and blue dotted lines depicts the three sub-networks participating in the layer cascading.

The output from the shared BiGRU layer $(BiGRU(x_1{\mathrm{:n}}))$ is fed to a shared attention layer whose output (*SAtt*) is passed to the independent dense layers of the three subnets. The rest of the information flow is similar to SPANMLC. Figure 4 depicts the overall architecture of the suggested framework.

*Calculation of loss* The framework’s total loss $(L(\Omega ))$ can be realized using the same loss function as SPANMLC.

#### Multitask 4

**Figure 5:**
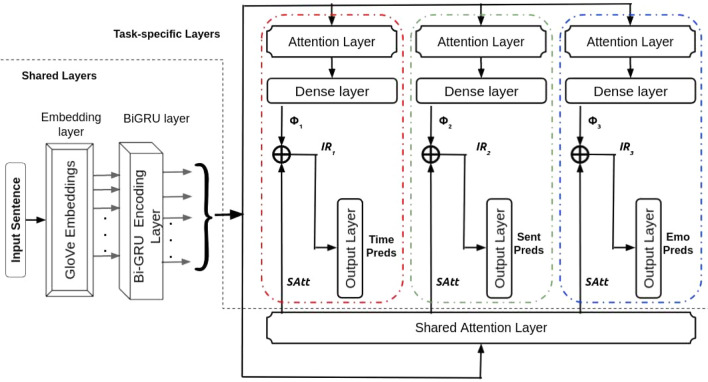
Architecture of shared-private attentive network without multi-layer cascades. Colour coded red, green and blue dotted lines depicts the three independent sub-networks.

To investigate the impact of having *private attention layers* in the overall architecture, we remove the cascading of layers in Fig. 1 (of the main file) by restricting the flow of intermediate output between subnets. The architecture for Multitask 4 (shown in Fig. 5)is similar to the SPANMLC except that we stop the flow of information among the subnets by not allowing a subnet’s intermediate representation to flow to the next subnet.

We first obtain $\Phi _{{1}}$, $\Phi _{{2}}$ and $\Phi _{{3}}$ following the equations (1), (2) and (3) respectively. Then, the intermediate representations are realized as follows:

15 $$\begin{aligned} IR_{{1}}= & {} [\Phi _{{1}};SAtt] \end{aligned}$$

16 $$\begin{aligned} IR_{{2}}= & {} [\Phi _{{2}};SAtt] \end{aligned}$$

17 $$\begin{aligned} IR_{{3}}= & {} [\Phi _{{3}};SAtt] \end{aligned}$$

Here, we compute the intermediate representation (IR_i_) of each sub-network by linearly concatenating the outputs from that particular subnet’s dense layer ($\Phi$_i_) and the shared attention layer (*SAtt*) only.

*Calculation of loss* The framework’s total loss $(L(\Omega ))$ is similar to Multitask 1.

### Results and discussion

**Figure 6:**
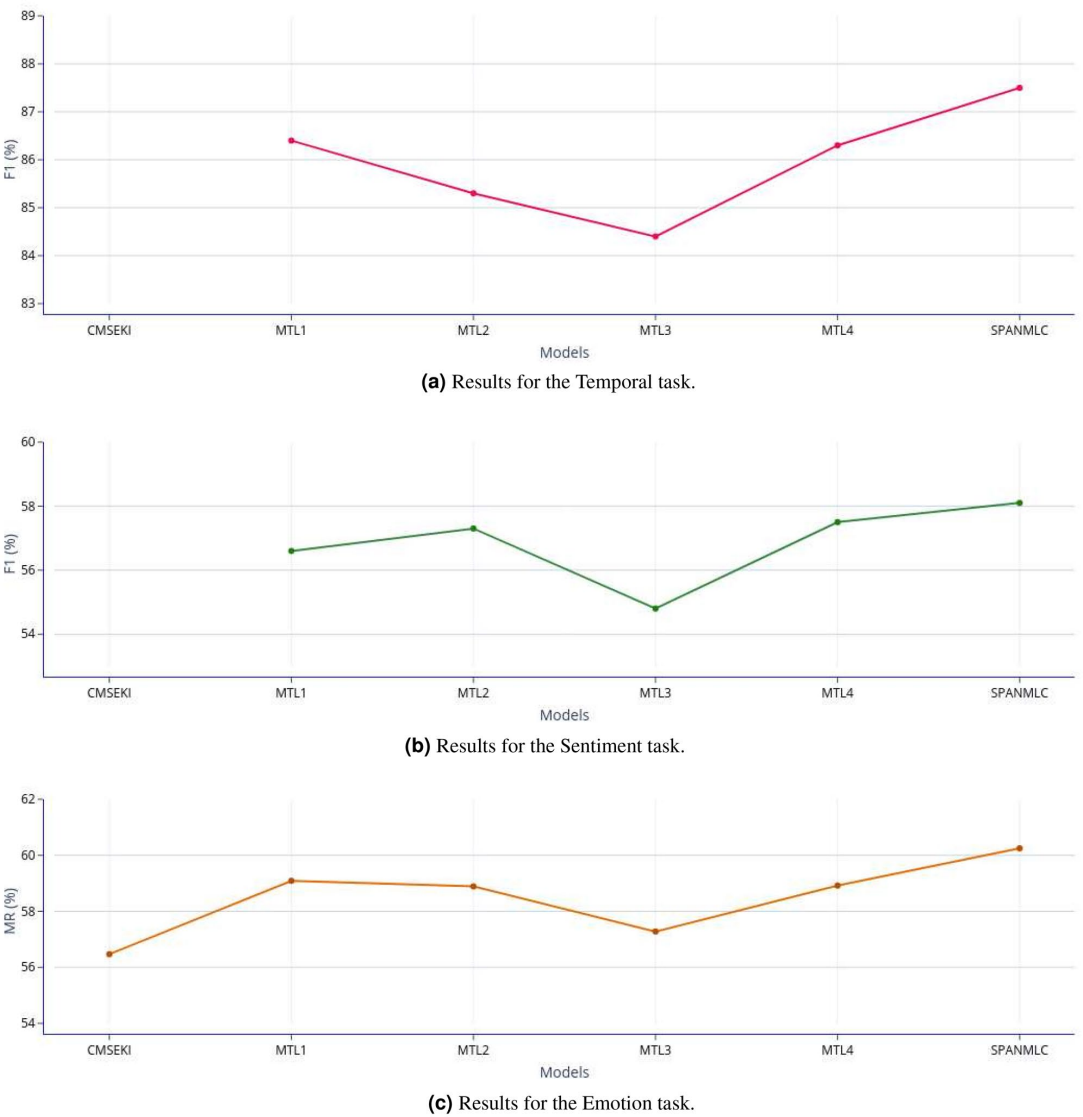
Graphical depiction of the scores from the various multitask learning (MTL) baselines and the proposed *SPANMLC* system.

The obtained results from the various experiments are reported in Table 5. Our proposed model (SPANMLC) outperformed the single-task system for the primary emotion recognition task by a significant improvement of 5.76% and 1.5% in terms of MR and sample-F1 metrics, respectively. The closest attained result on the emotion task is from the multitask baseline 1 (Multitask 1), which is 1.16% (MR) lower than the obtained score of SPANMLC. We also attained a 3.78% improvement over the state-of-the-art system, CMSEKI, on the Emotion recognition task, a multitask system involving three tasks. For the temporal orientation and sentiment detection tasks, SPANMLC outperforms others in terms of macro-F1 with scores of 87.5% and 58.1%, respectively. In terms of accuracy, our proposed system could not beat the state-of-the-art system on the sentiment task, which attained the top score of 65.33%. For the Temporal orientation task, our system top-scored with the accuracy of 87.5% but fell short by a small margin of 0.17% compared to the best-performing system for temporal orientation detection. We have also depicted the results for each task (*temporal*, *sentiment* and *emotion*) graphically in Fig. 6. Specifically, we considered the results from the various multitask baselines, and the proposed multitask *SPANMLC* system to plot the task-wise graphs in Fig. 6a–c. We plot the F1-scores for the *temporal* and *sentiment* task and the Mean Recall (MR) for the *emotion* task in the graphs.

**Table 5:** Evaluation results of tenfold cross-validation.

Tasks	Temporal task		Sentiment task		Emotion task	
Models	Acc. (%)	F1 (%)	Acc. (%)	F1 (%)	MR (%)	s-F1 (%)
**Single-task baselines**						
Single-task	**89**.**11**	87.3	62.29	57.4	54.49	50.8
**Multitask baselines**						
CMSEKI*^6^	–	–	**65**.**33**	–	56.47	51.8
Multitask 1*^18^	88.14	86.4	61.52	56.6	59.09	51.6
Multitask 2*^40^	87.36	85.3	61.95	57.3	58.89	51.6
Multitask 3	86.44	84.4	61.14	54.8	57.28	50.7
Multitask 4	87.89	86.3	61.82	57.5	58.92	51.5
**Proposed multitask approach**						
SPANMLC	88.94	**87**.**5**	62.31	**58**.**1**	**60**.**25**	**52**.**3**

Values in bold are the maximum scores attained.

MR: Mean Recall, s-F1: samples-F1.

The baselines marked with * are the state-of-the-art methods and the results reported in the table are directly fetched from the cited work.

Non-reported results for any particular metric is depicted as ’–’ in the table.

Below are some sample pre-processed sentences from our test set and predictions on various tasks from the different models:

- Sentence 1: *“the sexual abuse i endured from* $<name>$ *in* $<address>$ *all the migraines an sleep deprivation he tortured me with .”*
  Incorrect Emotion predictions from the Multitask 1 and Multitask 2 systems: Sorrow and Information respectively.
  Correct Emotion prediction from SPANMLC: Blame
- Sentence 2: *“my mind is messy .”*
  Incorrect Sentiment prediction from the multitask baselines: Neutral
  Correct Sentiment prediction from SPANMLC: Negative
- Sentence 3: *“i actively despise the person i am .”*
  Incorrect Temporal Orientation prediction from the non-cascaded multitask systems: Future
  Correct Temporal Orientation prediction from the cascaded systems (multitask 3 and SPANMLC): Present

We observe that correct classifications are highly biased towards over-represented classes in the dataset, such as *information, instructions, hopefulness*. Also, emotion classes consisting primarily of explicit instances (*forgiveness, thankfulness, love*) tend to be attended well by all the multitasking systems. Detailed qualitative analysis is discussed in the appendix.

**Figure 7:**
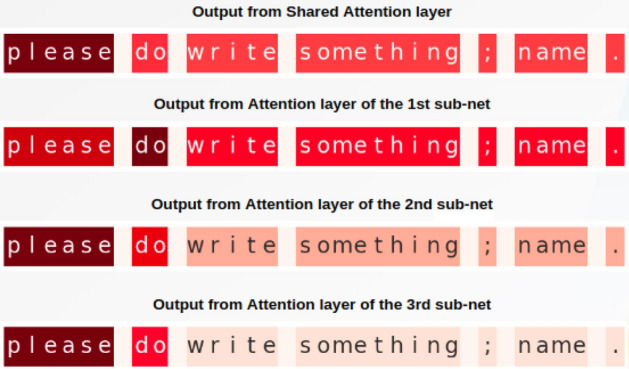
Attention visualization of the representations of a sentence (*Please do write something; name.*) from the different attention layers.

We use heatmaps to illustrate the sentence-level attention vectors from the various attention layers in our proposed system in Fig. 7. The color intensity denotes the importance of the words or phrases. We observe that the shared attention layer focuses significantly on all the words in the sentence. In contrast, the focus becomes more concentrated towards relevant terms as we progress through the subnets, thus improving model efficiency.

The results for the proposed system in Table 5 are statistically significant when tested against the null hypothesis (*p* value < 0.05). We performed Student’s t-test for the test of significance.

#### Comparison with state-of-the-art systems

In this work, we build a couple of multitasking baselines based on two popular state-of-the-art approaches: a) multitask approach for emotion, sentiment, and intensity prediction^41^ b) multitask framework using multi-network cascades^57^. *Multitask 1* and *Multitask 2* (described in “Baselines” section are necessary modifications to the architectures to fit our scenario. The reported results in Table 5 depict that our proposed model outperforms both the considered state-of-the-art baselines significantly on all three tasks (specifically, by 1.16 and 1.36 MR points on the emotion task, respectively). Most importantly, our approach outperforms the multitask state-of-the-art system^6^ that introduced the CEASE-v2.0 dataset by 3.78% MR points on the primary task of Emotion recognition.

#### Effectiveness of the auxiliary tasks

To investigate the impact of each of the auxiliary tasks on the primary task, we develop two separate multitask systems similar to SPANMLC (having two subnets instead of three), each involving a pair of auxiliary tasks and primary task: (a). Multitask^Time-Emo^ - models temporal orientation detection and emotion recognition, (b). Multitask^Sent-Emo^ - models sentiment detection and emotion recognition. The results indicate that the sentiment task (Multitask^Sent-Emo^ attains the MR of 59.56%) contributed more to the learning process of the emotion task in compared to the temporal orientation task (Multitask^Time-Emo^ attained MR of 58.32%). However, the best results (MR of 60.27%) are achieved when all the three tasks are involved in the learning simultaneously.

### Qualitative analysis

Table 6 shows the qualitative analysis of predictions on some sample test instances and observe the differences in performances when we train: (a) a system for each task independently (single-task systems) (b) a pair of primary task (Emotion detection), and secondary task (Temporal Orientation/Sentiment detection) together (two tasks systems); (c) all the three tasks together (proposed multitask system).

**Table 6:** Comparison among the performances of the single-task systems, dual multitask systems and the proposed system through qualitative analysis.

Sentence	Model	Temp. orient.	Sentiment	Emotion
Do not miss me	Single-task	Present	**Neutral**	Inf.
	Multitask^Time-Emo^	**Future**	–	Inf., **Instr.**
	Multitask^Sent-Emo^	–	**Neutral**	Inf.
	SPANMLC	**Future**	**Neutral**	**Instr.**
I am leaving, to much stressed out	Single-task	Present	Neutral	**Inf.**
	Multitask^Time-Emo^	**Past**	–	**Inf.**
	Multitask^Sent-Emo^	–	**Negative**	**Inf.**
	SPANMLC	**Past**	**Negative**	**Sor., Inf.**
I am very happy to have seen for myself the special person you have become	Single-task	Present	**Positive**	Inf.
	Multitask^Time-Emo^	**Past**	–	Inf.
	Multitask^Sent-Emo^	–	**Positive**	Inf.
	SPANMLC	**Past**	**Positive**	**Happ.**
For why persevere to exist if it is only to please the government	Single-task	Present	**Neutral**	Inf.
	Multitask^Time-Emo^	**Future**	–	Inf., **Instr.**
	Multitask^Sent-Emo^	–	**Neutral**	Inf.
	SPANMLC	**Past**	**Neutral**	**Instr.**
My fondes dreams are shattered because of a petty official; because of bad planning I planned too much. I decide to end it	Single-task	Present	**Neutral**	Inf.
	Multitask^Time-Emo^	Future	–	**Sor.**, Inf.
	Multitask^Sent-Emo^	–	**Negative**	Inf.
	SPANMLC	**Past**	**Negative**	**Sor.**

Texts in bold indicates actual or correct labels.

*Inf.* information, *Instr.* instructions, *Sor.* sorrow, *Happ.* happiness.

We observe from the table that labels for sentiment are predicted correctly by almost all the systems consistently, indicating that the sentiment detection task is more straightforward than the other two tasks. Some level of inconsistency is noticed in predictions for Temporal Orientation between the single-task model and Multitask^Time-Emo^ indicates that learning another task jointly along with the primary task impacts the overall training process.

We can observe how temporal information has helped the multitask systems correctly identify the associated emotion from the first and third instances. In the second sentence, we notice how the temporal information-assisted SPANMLC model can correctly identify the correct emotion labels (Sorrow, Information), considering the complete contextual information of that particular instance. On the other hand, the single-task system is only able to detect the *Information* label correctly because it focuses on the first part of the sentence ignoring the remaining contextual information.

We observe that in most of the cases, unlike the single-task system, the Multitask^Time-Emo^ system has predicted the temporal orientation label correctly, indicating a positive correlation among the emotion and temporal orientation tasks. For the emotion task, we observe that a chunk of the predictions revolves around two classes, *Information* and *Instructions*. The reason is that these two classes have a higher number of samples in the dataset compared to the rest of the classes. However, our proposed multitask system can identify correct classes for many instances where the single-task systems or dual multitask systems fail to identify the precise emotion class correctly.

We also observe that correct classifications are highly biased towards over-represented classes in the dataset, such as *information, instructions, hopefulness*. Also, emotion classes consisting primarily of explicit instances (*forgiveness, thankfulness, love*) tend to be attended well by all the multitasking systems.

Suicidal thoughts and/or actions in the *past* time perspective are unquestionably the most serious of the characteristics that we observe from qualitative analysis of our results.

#### Error analysis

Below are some sample sentences where our proposed system failed to make correct predictions:

- Sentence 1: *“This is a large part of the way that the world has gone wrong, the endless defining and dividing of things, micro-sub-categorization, sectarianism. ”*
  Our proposed approach predicted the emotion as *Sorrow* which is a bit of overestimation considering the underlying negative tone of the sentence. Most of the multitask baselines correctly predicted the emotion as *Information*.
- Sentence 2: *“Once deciding on suicide (I do not regard it as a sin, as Westerners do), I worked out the least painful means of carrying it out.”*
  Both the SPANMLC and Multitask 3 systems which are based on layer cascading, have predicted the sentiment as *Negative*. In contrast, the actual label is *Neutral* as correctly predicted by the other multitask baselines.
- Sentence 3: *“I guess I am crying because I am sorry mom and dad, but I am happy that I will be in heaven and no more attacks.”*
  The predicted TPO from the SPANMLC system is *Past*, whereas the correct prediction should have been either *Present* or *Future* as there are traces of both present and future time perspectives in the sentence. The Multitask 1 system correctly predicted the TPO as *Present* whereas both the Multitask 3 and Multitask 4 systems predicted *Future*.

To summarize, the majority of the misclassifications for the sentiment task are attributed to two reasons: a) Implicit instances (sentences where the sentiment is not expressed directly); b) presence of negative polarity word(s) in *neutral* instances. For the Emotion task, our proposed approach finds difficulty in making correct predictions due to the limitations above of the sentiment task along with the critical issue of data skewness (uneven distribution of instances over the various emotion classes). When a verb’s tense is mainly past, but the tweet is oriented in the present, the present tweets are misclassified as past tweets. Future-oriented tweets are frequently misclassified as past-oriented tweets. These misclassifications are caused by the past tense or the tweet being a compound phrase with an independent sentence relating to the past orientation.

### Challenges confronted while performing this study

This section discusses the various troubles confronted while performing this research. We categorize the challenges across three aspects:

- *Skewness and size of the dataset* The small size of the dataset is a major hindrance towards exploiting the richness of the various deep neural components employed in the proposed architecture. Furthermore, the dataset is highly skewed over the emotion classes, making the task of emotion detection even more challenging. Some classes are severely under-represented (abuse, pride, fear, etc.), while few classes are highly over-represented (information, hopelessness, instructions, etc.).
- *Data annotation* The weak annotations for the temporal categories and the emotion-to-sentiment mapped labels comes with their own challenges. While the mapping of the sentiment categories based on the associated emotion is correct for most of the instances, we observe certain anomalies in the resultant dataset. Some instances in the non-emotive classes such as *Information* and *Instructions* have a negative undertone yet marked as *Neutral*, since non-emotive classes are theoretically^27^ associated to bear *Neutral* polarity. Manual analysis of instances from the CEASE-v2.0 dataset has shown that few sentences contain information from varying time frames, where an event from the past was being referred the author was still feeling whose effect. In such instances, for the temporality labels obtained through the predictions of a weak classifier, we observe biasedness in the assignment of labels towards the over-represented *past* temporal category and somewhat towards *future* too.
- *Finding relevant background work* Locating relevant background work to validate the research findings has been a challenge as suicidal research with empirical methods using natural language processing approaches is quite scarce.

## Conclusion

In this work, we have proposed a multitask system to identify temporal orientation, sentiment detection, and multi-label emotion recognition from suicide notes. We have developed a shared-private attentive model with multi-network cascades for effective knowledge sharing among the various tasks. We compare the performance of our proposed multitask model with (a). the task-specific single-task systems (b). A state-of-the-art system (c). a couple of developed multitask baselines (d). a couple of state-of-the-art architectures. The proposed multitask system outperformed all the single-task and multitask baselines on the primary task of emotion recognition. It scored top on the emotion task with an MR score of 60.25% which is a 3.78 points improvement over the state-of-the-art system proposed in^6^. Our proposed approach also significantly improved performance (+5.76 points) than the single-task emotion system. The external knowledge-infused baseline system scored moderately better than our system in the sentiment task; however, our proposed model obtained an overall best F1 score of 87.5% but could not beat the best-attained accuracy from the single-task system a narrow margin of 0.17% on the temporal orientation task. We observe, both empirically and qualitatively, that concepts of temporality and sentiment are positively correlated with the task of emotion recognition when learned jointly.

We learn that analysis of suicide notes has much potential for multi-faceted research from various perspectives through this work. One possible drawback of this study is that the notes in the CEASE-v2.0 corpus were written at different times. Furthermore, the corpus exclusively contains English phrases. In the future, we would like to look at how language evolution influences the linguistic traits that distinguish recent notes. We would also want to eliminate another possible limitation of this study: considering the weak sentiment and temporal labels while building the proposed system, which may have performed better if manual annotations were considered for the tasks described above. We intend to extend this study with similar correlated tasks for multiple aspects of suicidal research, such as Mental Trauma, PTSD, Depression, etc. We will plan to use accessible social-media data to routinely access suicidal risk factors and time-varying actions, which may be a helpful channel for new research into public health issues.

### Ethics declarations

Our resource creation utilizes publicly available CEASE-v2.0^6^ benchmark suicide notes dataset. We have followed the policies of using the data and have not harmed any copyright issues. We intend to make the codes and note-level personality annotations available (with reference note-ids only and not the notes themselves) only after filling and signing an agreement declaring that the data will be used only for research purposes. This study was also reviewed by our Institutional Review Board (IRB) and was found that it did not require ethical review or supervision.

## Data Availability

The codes and data will be made available at https://www.iitp.ac.in/~ai-nlp-ml/resources.html#SPANMLC. The authors wish to confirm that there are no known conflicts of interest associated with this publication.
